# Polysaccharide mycophenolate-based nanoparticles for enhanced immunosuppression and treatment of immune-mediated inflammatory diseases

**DOI:** 10.7150/thno.52891

**Published:** 2021-01-26

**Authors:** Yuce Li, Yuchen Lou, Yu Chen, Jing Yang, Danqi Li, Biling Jiang, Jiajia Lan, Jingjing Wen, Yangxue Fu, Yamin Zhang, Juan Tao, Jintao Zhu

**Affiliations:** 1 Key Laboratory of Material Chemistry for Energy Conversion and Storage (HUST) of Ministry of Education, School of Chemistry and Chemical Engineering, Huazhong University of Science and Technology (HUST), Wuhan 430074, China.; 2 Department of Dermatology, Union Hospital, Tongji Medical College, HUST, Wuhan 430022, China.

**Keywords:** mycophenolate, polysaccharide, immunosuppression, psoriasis, immune-mediated inflammatory diseases

## Abstract

Immune-mediated inflammatory diseases (IMIDs) are characterized by immune dysregulation and severe inflammation caused by the aberrant and overactive host immunological response. Mycophenolic acid (MPA)-based immunosuppressive drugs are potential treatments for IMIDs because of their mild side-effect profile; however, their therapeutic effects are limited by the high albumin binding rate, unsatisfactory pharmacokinetics, and undefined cellular uptake selectivity.

**Methods:** Polysaccharide mycophenolate was synthesized by conjugating MPA molecules to dextran (a typical polysaccharide widely used in drug delivery) and encapsulated extra free MPA molecules to fabricate MPA@Dex-MPA nanoparticles (NPs). The efficacy of these NPs for mediating immunosuppression and treatment of IMIDs was evaluated in imiquimod-induced psoriasis-like skin inflammation in Balb/c mice, a representative IMID model.

**Results:** The MPA@Dex-MPA NPs exhibited high MPA loading efficiency, low albumin binding rates, and sustained MPA release, resulting in improved pharmacokinetics *in vivo*. Compared to free MPA, MPA@Dex-MPA NPs induced more robust therapeutic effects on IMIDs. Mechanistic studies indicated that MPA@Dex-MPA NPs were primarily distributed in dendritic cells (DCs) and significantly suppressed the overactivated DCs *in vivo* and *in vitro*. Furthermore, the recovered DCs rehabilitated the IL-23/Th17 axis function and significantly ameliorated imiquimod-induced psoriasis-like skin inflammation. Importantly, MPA@Dex-MPA NPs showed favorable safety and biocompatibility* in vivo*.

**Conclusion:** Our results indicated the polysaccharide mycophenolate-based NPs to be highly promising for IMID treatment.

## Introduction

Immune-mediated inflammatory diseases (IMIDs), including inflammatory bowel disease, psoriasis/psoriatic arthritis, rheumatoid arthritis (RA), ankylosing spondylitis, and systemic lupus erythematosus (SLE), are a class of diseases characterized by immune dysregulation and severe inflammation 1-6. IMIDs are usually chronic, debilitating, and cause end-organ damage and even death 7, 8. They also contribute to neurodegeneration, cancer, long-term pain, and some psychiatric and mental health disorders 9, 10. Moreover, patients with one type of IMID are at a higher risk to suffer from other IMIDs during their lifetime; for example, about 1/5 of psoriasis patients also suffer from psoriatic arthritis 11-13.

Although different types of IMIDs are clinically heterogeneous and ostensibly unrelated due to their diverse symptoms, they share common pathogenesis of the aberrant and overactive host immunological response 14-18. Typically, over-activation of antigen-presenting cells (APCs) such as dendritic cells (DCs) and the subsequent immune response, such as secretion of cytokines and activation of T lymphocytes, are frequently observed in IMID cases 19. For example, psoriasis, a chronic IMID with manifestations of erythematous plaques and silvery scale 20, is believed to be associated with the accumulation of immunostimulatory myeloid DCs and over-activation of Interleukin-23 (IL-23)/T_h_17 axis 21-23. IL-23 is mainly produced by DCs and maintains the development of T_h_17 cells, which can produce various cytokines, including IL-17A, IL-17F, and IL-22. Aberrant expressions of IL-17 and IL-22 induce epithelial pathology, including keratinocyte proliferation as well as a series of complex feedback loops involving keratinocytes, neutrophils, mast cells, T cells, and DCs, resulting in the development of psoriasis 24-26.

Immunosuppressive drugs are effective in inducing immunosuppression and treating IMIDs 27-29. Mycophenolic acid (MPA), the active metabolite of commercial prodrug mycophenolate mofetil (MMF), is an effective immunosuppressive drug for organ transplantations and IMIDs, e.g., psoriasis 30-32. Compared with other clinically available immunosuppressive drugs (e.g., methotrexate, cyclosporine, and azathioprine), MMF shows a favorable side-effect profile, which is more acceptable for patients 33-35. When orally administrated, MMF is rapidly absorbed in the upper gastrointestinal tract and hydrolyzed into active MPA, which can reversibly inhibit inosine 5'-monophosphate dehydrogenase (IMPDH) that plays an essential role in the synthesis of guanine nucleotides 30. IMPDH is mainly expressed in activated lymphocytes, which are heavily dependent upon the *de novo* pathway. Therefore, MPA could induce a strong cytostatic effect on activated T and B lymphocytes 31, 36 and also showed cytostatic effects on other types of immune cells, some of which were IMPDH-independent 32, 37. For example, MPA was reported to induce immature DCs to tolerogenic type, inhibiting the expression of surface proteins (e.g., CD80, CD86 and major histocompatibility complex) and subsequently lead to T-cell-mediated immunosuppression 38-41. Furthermore, MPA-treated DCs could induce Th2-type allogeneic CD4+ T cells and Treg cells 42.

Despite the advantages of MPA derivates for immunosuppression mentioned above, they are not the first-line drug for IMIDs (e.g., psoriasis) due to their insufficient therapeutic effect and relatively long onset time (usually several months) 43-45. This can be ascribed to the following reasons: (1) after administration, a range of 97% to 99% of MPA binds to the plasma albumin at clinically relevant concentrations, resulting in decreased bioavailability 46; (2) MPA displays non-linear pharmacokinetics, with complex and large pharmacokinetic variability due to the diverse serum albumin levels, renal function, genetics, drug-drug interactions in patients, and a half-life of about 8-16 h following administration, requiring at least twice per day administration 30, 31, 43, 47; (3) there is no uptake selectivity in different cell types, significantly decreasing the delivery efficiency to the target immunocytes 48, 49.

Drug delivery systems with nano-scaled sizes provide a new opportunity to efficiently deliver MPA due to their unique properties to protect the drugs, target specific cells, and steadily release the payloads. Fahmy et al. developed several MPA-loaded nanoparticles (NPs) based on lipids and poly(lactic-*co*-glycolic acid) (PLGA) for the treatment of SLE 38, 50. These NPs could be internalized by DCs and suppressed the inflammatory responses, benefiting the treatment and survival of lupus-prone mice. However, these systems showed very low drug-loading efficiency (<1%, *w*/*w*) and short release duration (within several hours) because MPA was loaded through hydrophobic interactions or physical encapsulation. Therefore, it is imperative to develop drug delivery systems that would decrease the albumin binding rate, regulate pharmacokinetics, and mediate targeted delivery to desired immunocytes for a better therapeutic effect 51.

Herein, we developed polysaccharide mycophenolate-based NPs and evaluated their ability to mediate immunosuppression and treatment of IMIDs, using imiquimod (IMQ, a Toll-like receptor-7/8 agonist)-induced psoriasis-like skin inflammation as the model disease 52. In this system, MPA molecules were conjugated to dextran by ester bonds, which slowly dissociated by esterase to provide a sustained release and improved MPA pharmacokinetics. NPs of dextran-MPA conjugates (Dex-MPA NPs) were prepared through an ultrasonic emulsification and solvent evaporation approach. Moreover, additional free MPA molecules were encapsulated into the NPs (MPA@Dex-MPA NPs) during the emulsion-solvent evaporation process to provide enough initial concentration to shorten the onset time. The multiple hydroxyl groups in polysaccharides and formation of NPs endowed the NPs with negligible plasma protein adsorption. These NPs were administered intraperitoneally (IP) into the mice, and their *in vivo* therapeutic effects against psoriasis were evaluated by the Psoriasis Area and Severity Index (PASI) scores (an objective estimate system widely used for the measurement of severity of psoriasis in the clinic), hematoxylin and eosin (H&E) staining, and immunohistochemistry (IHC). The ability of MPA@Dex-MPA NPs to induce immunosuppression and the underlying mechanism were systemically investigated through detecting cellular internalization by specific types of immunocytes, maturation of immunocytes, and secretion of cytokines both *in vivo* and *in vitro*. The biosafety and pharmacokinetic profiles were also determined, indicating these polysaccharide mycophenolate-based NPs to be promising in treating IMIDs.

## Results and Discussion

### Preparation and characterization of MPA@Dex-MPA NPs

**Figure 1:**
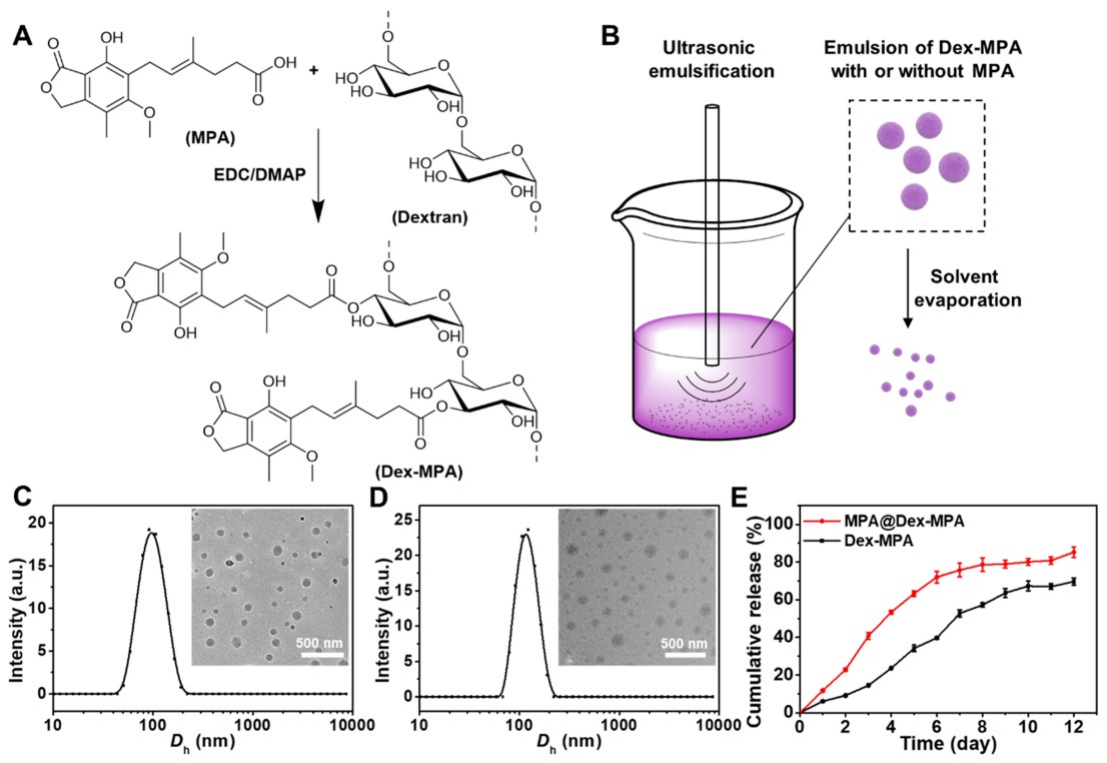
Preparation and characterization of MPA@Dex-MPA NPs. (A) Synthesis of Dex-MPA. (B) Schematic illustration showing the preparation of Dex-MPA NPs or MPA@Dex-MPA NPs. The mixture of Dex-MPA solution in CHCl_3_ (10 mg/mL, 5 mL) with or without free MPA (15 mg) and PVA aqueous solution (3 mg/mL, 50 mL) was emulsified using ultrasound, followed by thorough evaporation of CHCl_3_ under aseptic conditions. NPs were obtained after repetitive centrifugation and resuspension. (C, D) Hydrodynamic diameter (*D*_h_) and representative TEM images (inset) of Dex-MPA NPs (C) and MPA@Dex-MPA NPs (D). (E) *In vitro* MPA release from the NPs. Data are presented as mean ± SEM (*n* = 3).

Dextran-MPA conjugate (Dex-MPA) was synthesized by esterification between the carboxyl group in MPA and hydroxyl groups in dextran (**Figure 1**A). The chemical structure of Dex-MPA was confirmed by ^1^H NMR (**Figure S1** in the Supporting Information), and all peaks were well assigned. The integrations for peaks of anhydroglucose residues were less than theoretical values due to the formation of self-assembled structures in organic solvents, since anhydroglucose residues contain many hydroxyl groups, so that they have relatively low solubility and may form strong hydrogen bonds in organic solvents 53. Therefore, we quantified the amount of MPA in Dex-MPA by UV-vis spectroscopy (**Figure S2**). MPA content was determined to be 63.5%, indicating ~80.3% of anhydroglucose residues were conjugated with one MPA molecule and was close to the feeding ratio (1:1 in the molar ratio of MPA to anhydroglucose residue). The resultant Dex-MPA conjugate was utilized to prepare Dex-MPA NPs or MPA@Dex-MPA NPs through the ultrasonic emulsification and solvent evaporation method (Figure 1B). The MPA loading contents of both NPs are presented in **Table 1**. Compared with traditional drug loading methods through encapsulation or hydrophobic interactions between drug and carrier, the conjugated Dex-MPA showed very high drug loading content (>60%), which is one of the advantages of conjugated drugs. MPA in MPA@Dex-MPA NPs includes two parts, *i.e.*, the conjugated MPA (50.49%) and the loaded MPA (20.50%). Due to the excellent compatibility between MPA and Dex-MPA, the loading efficiency of MPA was higher than 85%.

We examined the morphologies and size distributions of both NPs by transmission electron microscopy (TEM) and dynamic laser scattering (DLS). Both Dex-MPA NPs and MPA@Dex-MPA NPs displayed sphere shapes and a similar average hydrodynamic diameter (*D*_h_) of ~100 nm (Figure 1C, D), indicating that the loading of free MPA did not influence the NP formation. Since polymers containing multiple hydroxyl groups (e.g., polysaccharides) were reported to decrease plasma protein adsorption 54, we determined the protein adsorption of MPA@Dex-MPA NPs by incubating them with bovine serum albumin (BSA) at various concentrations for different periods (**Figure S3**). MPA@Dex-MPA NPs showed minimal protein adsorption (<20 µg/mg) at all tested concentrations and all incubation times. These results indicated that MPA@Dex-MPA NPs could protect the MPA from adsorption by plasma proteins, which would benefit the bioavailability of MPA by systemic administration.

As the drug release profile was strongly related to the pharmacokinetics *in vivo*, we investigated the MPA release profile from the NPs *in vitro* by a dialysis method at 37 °C (Figure 1E). Dex-MPA NPs maintained gradual release for around 10 days to reach a final ~70% cumulative release ratio. In the first three days, the release rate was relatively slow because the NPs formed compact structures allowing hydrolysis and release of only MPA molecules on the surface. The release rate was significantly accelerated from day 4. A possible explanation for this phenomenon is that the swelling and partial disassembling of the NPs resulted in a looser structure so more MPA molecules could be hydrolyzed and released. The structure of released MPA was confirmed by HPLC-MS (**Figure S4**). In comparison, MPA@Dex-MPA NPs displayed a faster release behavior with more than 80% of MPA molecules released within eight days. The faster release was caused by the rapid release of encapsulated MPA molecules and facilitated hydrolysis of Dex-MPA due to the loose structure of MPA@Dex-MPA after the release of encapsulated MPA. This result indicated that the MPA@Dex-MPA NPs could provide a sustained drug release profile with a higher initial concentration of MPA compared with Dex-MPA NPs and shorten the onset time.

### Therapeutic effects of MPA@Dex-MPA NPs against IMIDs

**Figure 2:**
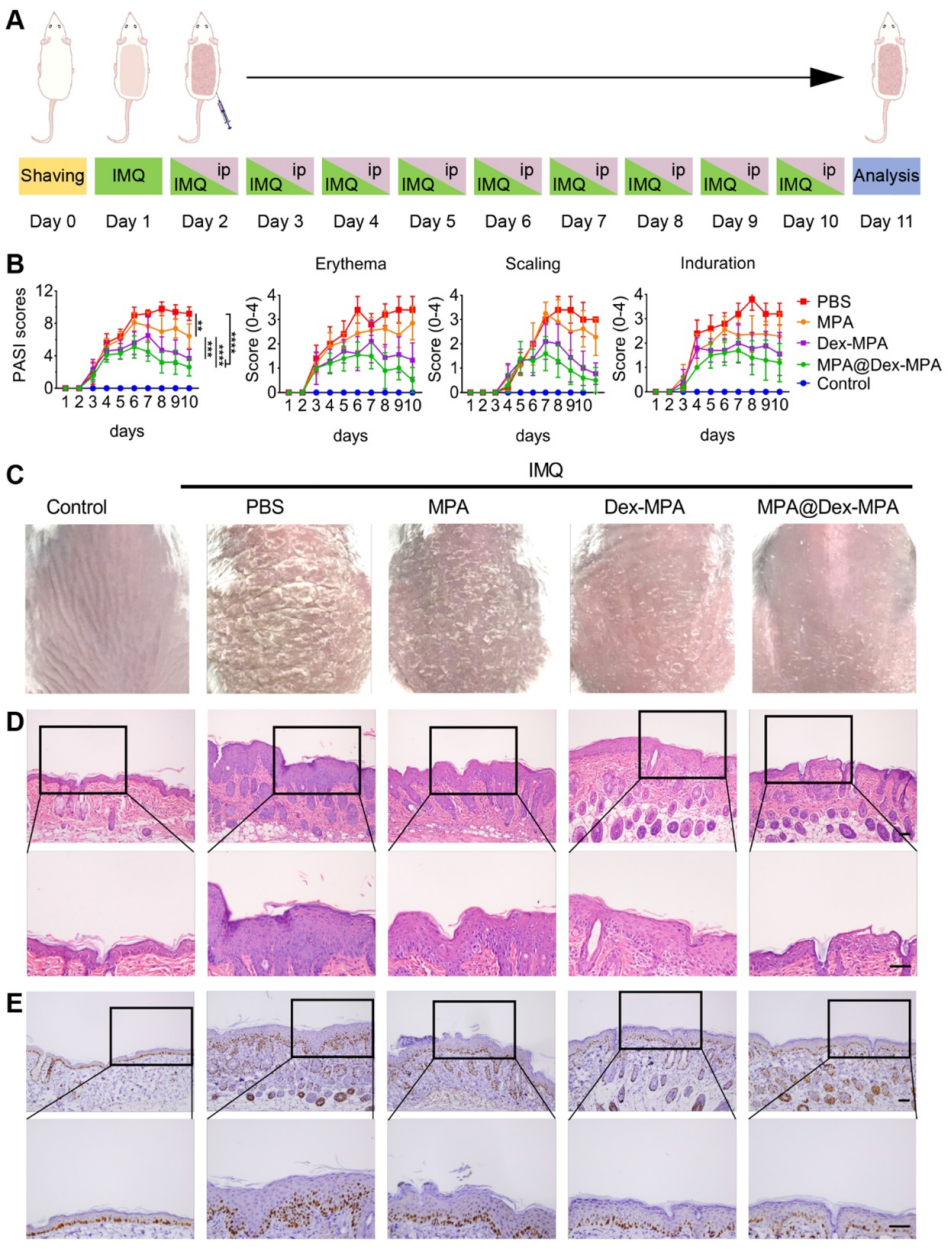
Therapeutic effects against IMIDs by different treatments. (A) I*n vivo* experimental protocol. Seven-week-old Balb/c mice were treated with IMQ daily on the shaved back skin for ten consecutive days, followed by IP injection of PBS, MPA solution, Dex-MPA NPs, or MPA@Dex-MPA NPs at 10 mg/kg/day of MPA equivalent for the following nine days, as indicated. (B) Daily PASI score curve, calculated by scores of erythema, scaling, and induration, ranges from 0 to 4. Data are presented as mean ± SEM (*n* = 5; *p < 0.05, **p < 0.01, ***p < 0.001 and ****p < 0.0001, one-way ANOVA with post-hoc Tukey test). (C) Representative phenotypic photographs of back skin on day 11. (D, E) Histological images of H&E staining (D) immunohistochemistry (IHC) staining of Ki-67 (E) for the skin lesions. All scale bars (100 μm) in the right images apply to the left ones.

To evaluate the biological activity of MPA@Dex-MPA NPs, we investigated their therapeutic effects against IMIDs, using IMQ-induced psoriasis-like skin inflammation in Balb/c mice as the model disease 55-58. The treatments were performed as shown in **Figure 2**A. The disease severity was assessed daily using the PASI score during the treatment (Figure 2B). All groups showed increased PASI scores without significant differences in the first four days. PASI scores in both Dex-MPA NP and MPA@Dex-MPA NP groups started to show significant differences from the PBS and MPA solution groups since day 5 (*p* < 0.05 for Dex-MPA NPs and *p* < 0.01 for MPA@Dex-MPA NPs). The PASI score started decreasing on day 7 by the MPA@Dex-MPA treatment, attributed to a faster release and higher initial concentration of the encapsulated free MPA in MPA@Dex-MPA NPs, while treatment with Dex-MPA showed a delayed onset time (day 8) (Figure 1E). In contrast, treatment with free MPA solution did not show any significant therapeutic effect compared with the PBS group until day 8 (*p* < 0.05). After 9 days of treatment, the MPA@Dex-MPA group exhibited the best therapeutic effects with the lowest PASI scores and the most significant amelioration of typical disease manifestations, including erythema, scaling, and induration as displayed in the photographs (Figure 2C). In comparison, treatment with the free MPA solution yielded unsatisfactory therapeutic effects because the MPA dose used in our experiment (10 mg/kg/day) was far less than the clinically recommended dose (30-100 mg/kg/day) 59, 60.

We performed the pathological analyses of biopsy specimens from the back-skin lesions of mice on day 11 by H&E and Ki-67 IHC staining (Figure 2D, E). Epidermal hyperplasia and infiltration of inflammatory cells were the characteristic features of psoriasis. Treatment with MPA@Dex-MPA NPs significantly decreased the epidermal thickness and inflammatory cell infiltration to the control group level, indicating the favorable therapeutic effects (Figure 2D), whereas these parameters were less affected by Dex-MPA NPs and MPA treatments.

Ki-67 is a keratinocyte proliferation marker and is the main manifestation of epidermal hyperplasia and dermal infiltration 61. Compared with the significantly increased Ki-67 expression in the PBS group, Ki-67 expression in the MPA@Dex-MPA group was almost the same as normal tissues (control), indicating that MPA@Dex-MPA NPs could significantly reverse the IMQ-induced increase of keratinocyte proliferation (Figure 2E). Notably, although all groups were treated with the same equivalent of MPA (10 mg/kg/day), MPA@Dex-MPA NPs showed better therapeutic effects than Dex-MPA NPs, while MPA solution induced inadequate effects (Figure 2). Thus, IP injection of MPA@Dex-MPA NPs effectively ameliorated skin inflammation of psoriasis. We investigated the pharmacokinetics, internalization in different types of immunocytes, and the subsequent immune responses mediated by MPA@Dex-MPA NPs both *in vivo* and *in vitro* to better understand their superior therapeutic effect compared to other formulations.

### Pharmacokinetics of MPA@Dex-MPA NPs

**Figure 3:**
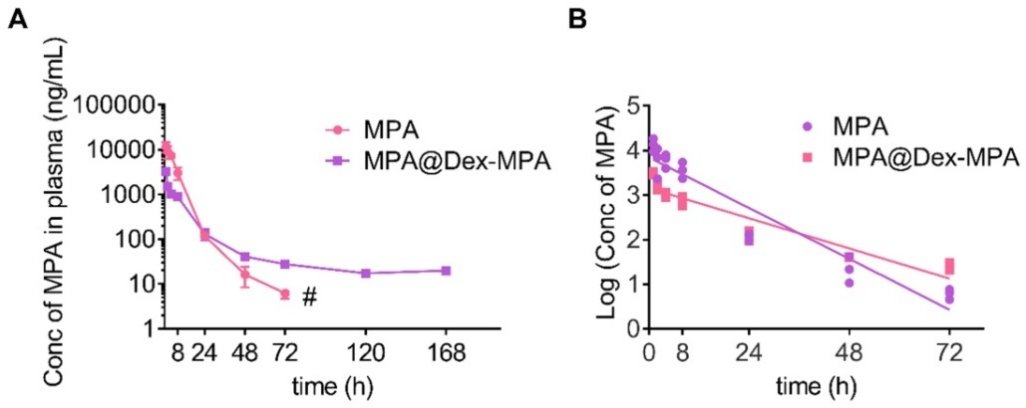
Pharmacokinetics of MPA *in vivo*. MPA or MPA@Dex-MPA NPs (10 mg/kg in MPA equiv.) were IP injected in rats, and the blood samples were collected at predetermined time points. Concentrations of MPA in plasma were measured by liquid chromatography-mass spectrometry (LC/MS). (A) Plasma concentration-time curve of MPA. (B) Log (plasma concentration of MPA)-time curve of MPA. **^#^** Undetectable in the following days.

The *in vivo* pharmacokinetics of MPA represents a key parameter of effective immunosuppression 47. Therefore, we studied the *in vivo* pharmacokinetics of MPA@Dex-MPA NPs to better understand their superior therapeutic effects on psoriasis compared to the MPA solution. Time-dependent plasma concentrations were measured post-IP injection of the MPA solution or MPA@Dex-MPA NPs (**Figure 3**), and relevant pharmacokinetic parameters were calculated (**Table 2**). For the MPA solution, terminal half-life (*t*_1/2_), *C*_max_, and area under the curve (AUC) were 14.7 ± 1.0 h, 11.5 ± 2.1 µg/mL and 79.8 ± 8.8 µg·h/mL, respectively, whereas for MPA@Dex-MPA NPs, these parameters were 24.64 ± 0.71 h, 3.2 ± 0.1 µg/mL, and 23.2 ± 0.8 µg·h/mL, respectively (Table 2). Although *C*_max_ and AUC in the MPA group were 3.7-fold and 4.8-fold higher than the MPA@Dex-MPA group, the plasma concentration of MPA was undetectable within three days in the MPA group, while the MPA@Dex-MPA group maintained a concentration at >15 ng/mL for more than 7 days. The prolonged plasma MPA in the MPA@Dex-MPA group could be attributed to the delayed and sustained release consistent with the *in vitro* drug release (Figure 1E). Furthermore, compared with the same dose in the MPA group, *t*_1/2_ of MPA in blood plasma was prolonged by 1.7-fold (*p* < 0.01) in the MPA@Dex-MPA group. The terminal half-life of a drug is usually regarded as the optimized interval for clinical administration, implying that as opposed to twice per day of free MPA, MPA@Dex-MPA NPs could be given only once per day for favorable therapeutic effect.

### Cell type-dependent delivery of MPA@Dex-MPA NPs *in vivo*

**Figure 4:**
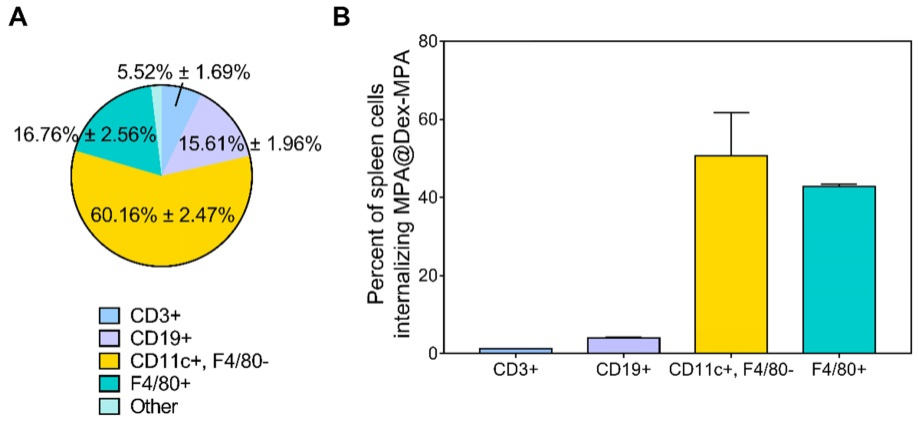
Cell type-dependent delivery of MPA@Dex-MPA NPs *in vivo*. Flow cytometry was employed to analyze the cells isolated from spleens of IMQ-treated Balb/c mice 2 h after IP injection of Nile Red-labeled MPA@Dex-MPA NPs. (A) Distribution of MPA@Dex-MPA NPs in different cell subsets. Data presented as the population percentage of one specific cell type among all the NP-positive cells; and (B) Percentages of NP-positive cells in different cell subsets in the spleen. Data presented as the percentage of NP-positive cells among the full population of one specific cell type. Results are expressed as mean ± SEM. CD3+, T cells; CD19+, B cells; CD11c+, F4/80-, DCs; F4/80+, macrophages.

The development of psoriasis is closely related to the functions of immune cells; however, due to the small molecule essence of MPA, there is no selectivity for its uptake in different cell types 30. We identified the main immune cell subsets in the spleen of IMQ-treated Balb/c mice by analyzing both the distribution of MPA@Dex-MPA NPs in different cell subsets (**Figure 4**A) and the NP-positive rate of each cell type (Figure 4B) by flow cytometry.

Nile Red-labeled MPA@Dex-MPA (NR-MPA@Dex-MPA) NPs were IP administered into IMQ-induced psoriasis mice and their distribution in different cell subsets was obtained by detecting the specific biomarkers of each cell subset among Nile Red-positive cells from spleens (Figure 4A). Data were presented as the population percentage of one specific cell type among all the Nile Red-positive cells. The results suggested that most of MPA@Dex-MPA NPs (60.16% ± 2.47%) were distributed to myeloid cells of the spleen and were primarily taken up by DCs (CD11c+, F4/80-). The rest of MPA@Dex-MPA NPs were internalized by macrophages (F4/80+; 16.76% ± 2.56%), B cells (CD19+; 15.61% ± 1.96%), T cells (CD3+; 5.52% ± 1.69%), and some unidentified cells.

To better understand the higher uptake of MPA@Dex-MPA NPs by DCs, we determined the NP-positive cell subsets in spleens of IMQ-induced psoriasis mice and examined the ability of each cell subset to internalize MPA@Dex-MPA NPs (Figure 4B). Data were presented as the percentage of Nile Red-positive cells among the population of one specific cell type. The DCs showed a slightly higher ability to internalize the MPA@Dex-MPA NPs (50.73% ± 8.92%) than the macrophages (42.75% ± 0.45%), possibly because DCs were the primary APCs taking up the small-size exogenous components 38, 62, 63. However, this could not be the main reason for the higher uptake of MPA@Dex-MPA NPs by DCs rather than macrophages (Figure 4A). A possible explanation could be that DCs, especially the subsets of dermal and plasmacytoid DCs (pDCs), were significantly increased in spleens of psoriasis mice 52. This rationale was supported by the smaller ratio of NP-positive DCs detected in spleens of normal mice (43.02% ± 1.75%) than the psoriasis mice (**Figure S5**). Moreover, although T and B lymphocytes are the main cell subsets in the spleen, only around 21% of NPs were found in the lymphocytes (Figure 4A) and less than 5% of either T or B lymphocytes internalized the NPs (Figure 4B), indicating that the immunosuppression and therapeutic effect of MPA@Dex-MPA NPs in psoriasis mice were not primarily mediated by direct suppression of T or B lymphocytes. MPA was generally considered to be more effective in inhibiting T and B lymphocyte proliferation than other cell types; it was more effective in inhibiting the type II IMPDH isoform (IMPDH-2), specifically expressed in lymphocytes, than the type I isoform (IMPDH-1), expressed in other cell types 37. However, recent studies indicated that MPA also affected other types of immune cells, such as monocytes and DCs 32, and some effects were independent of IMPDH 64.

### Enhanced immunosuppression by MPA@Dex-MPA NPs through DC suppression

**Figure 5:**
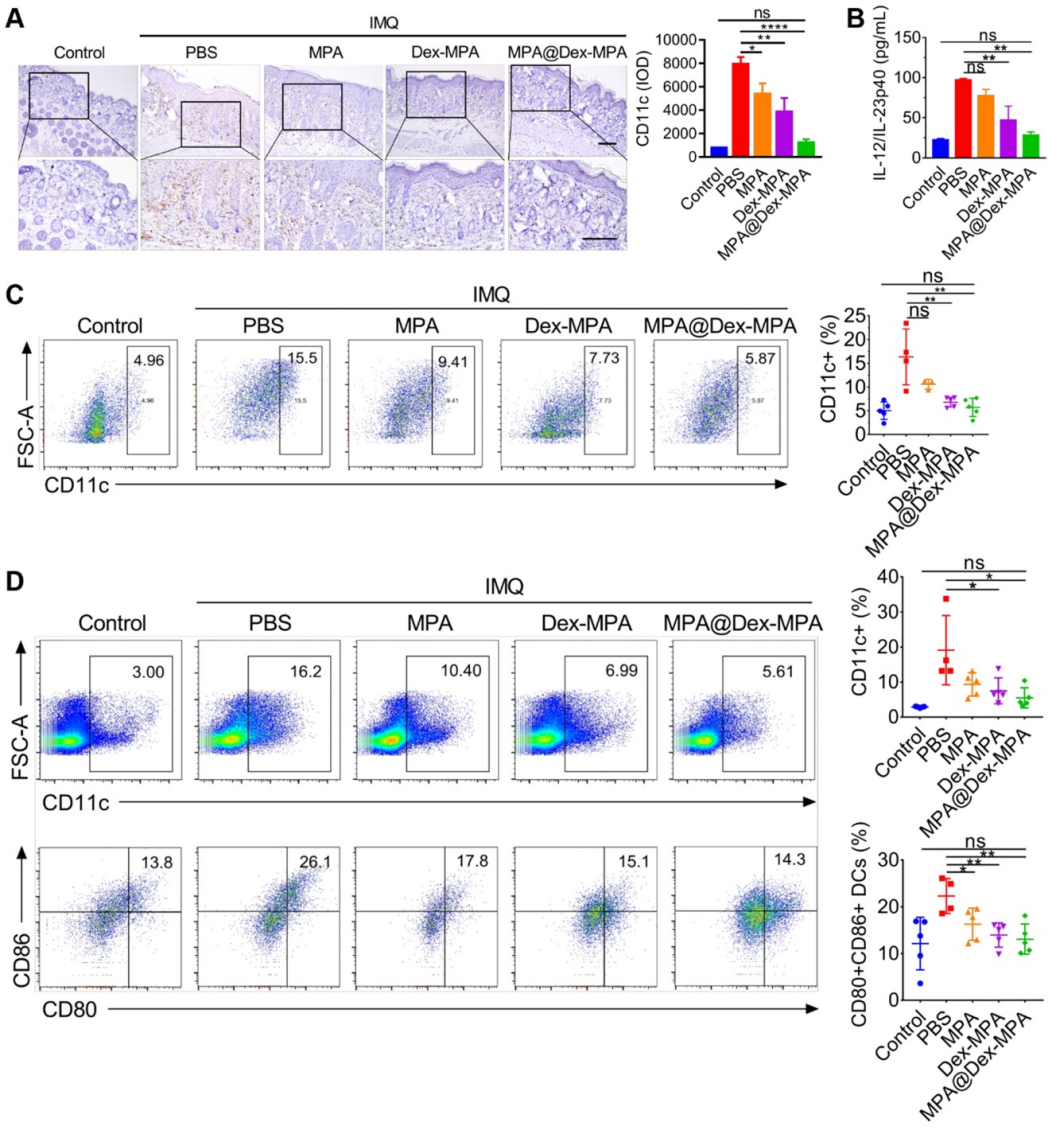
*In vivo* population, maturation, and cytokine secretion of CD11c+ DCs by different treatments. (A) CD11c IHC staining images and IHC result analysis represented by integral optical density (IOD) values of back skin lesion biopsy on day 11. All scale bars (100 μm) in the right images apply to the left ones. (B) ELISA for IL-12/IL-23p40 of serum samples collected from mice on day 11. (C, D) Flow cytometry of spleens and dLNs harvested on day 11. (C) CD11c+ cells in spleens. (D) CD11c+ cells and the maturated CD80+, CD86+ subset among CD11c+ cells in inguinal lymph nodes. Data are presented as mean ± SEM (*n* = 5; *p < 0.05, **p < 0.01, ***p < 0.001 and ****p < 0.0001; one-way ANOVA with post-hoc Tukey test).

Since DCs were the primary target of MPA@Dex-MPA NPs *in vivo*, we further examined the effects of MPA@Dex-MPA NPs on CD11c+ DCs and evaluated the systemic immune environment. The back skin lesions, spleens, and inguinal lymph nodes from each group were collected for a series of analyses after the treatments (**Figure 5**). We examined the infiltration of CD11c+ DCs into the dermis by IHC staining (Figure 5A). MPA@Dex-MPA NPs remarkably reduced CD11c+ DC population in the skin compared to the PBS group, suggesting that the therapeutic effects could be induced partially by ameliorating DCs infiltration into the skin. Subsequently, we collected sera of mice before sacrifice and evaluated each group's cytokine expression by enzyme-linked immunosorbent assay (ELISA) (Figure 5B). The level of proinflammatory cytokine IL-23 increased to about four-fold upon IMQ treatment (*p* < 0.01) while it almost reduced to the normal level after treatment with MPA@Dex-MPA NPs (*p* < 0.01). Furthermore, quantitative flow cytometry of spleens and draining lymph nodes (dLNs) demonstrated that CD11c+ DCs decreased by 62% in spleens (*p* < 0.01, Figure 5C) and 47% in dLNs (*p* < 0.05, Figure 5D) after treatment with MPA@Dex-MPA NPs compared with the PBS group, and showed no significant differences with the control group. Moreover, CD80 and CD86 (markers of activated DCs) expression by DCs in the dLNs of MPA@Dex-MPA group decreased by 42% (*p* < 0.01, Figure 5D) with no significant differences compared with controls, indicating that MPA@Dex-MPA treatment also inhibited DC maturation in IMQ-induced psoriasis model. Since DCs are the primary source of IL-23 in the IMQ-induced psoriasis murine model 15, 20, 65, reduced IL-23 expression was concordant with the decrease in CD11c+ DCs both in skin lesion and spleens. These results demonstrated that MPA@Dex-MPA NPs not only decreased the DC population but also suppressed the maturation and secretion of cytokines by DCs.

**Figure 6:**
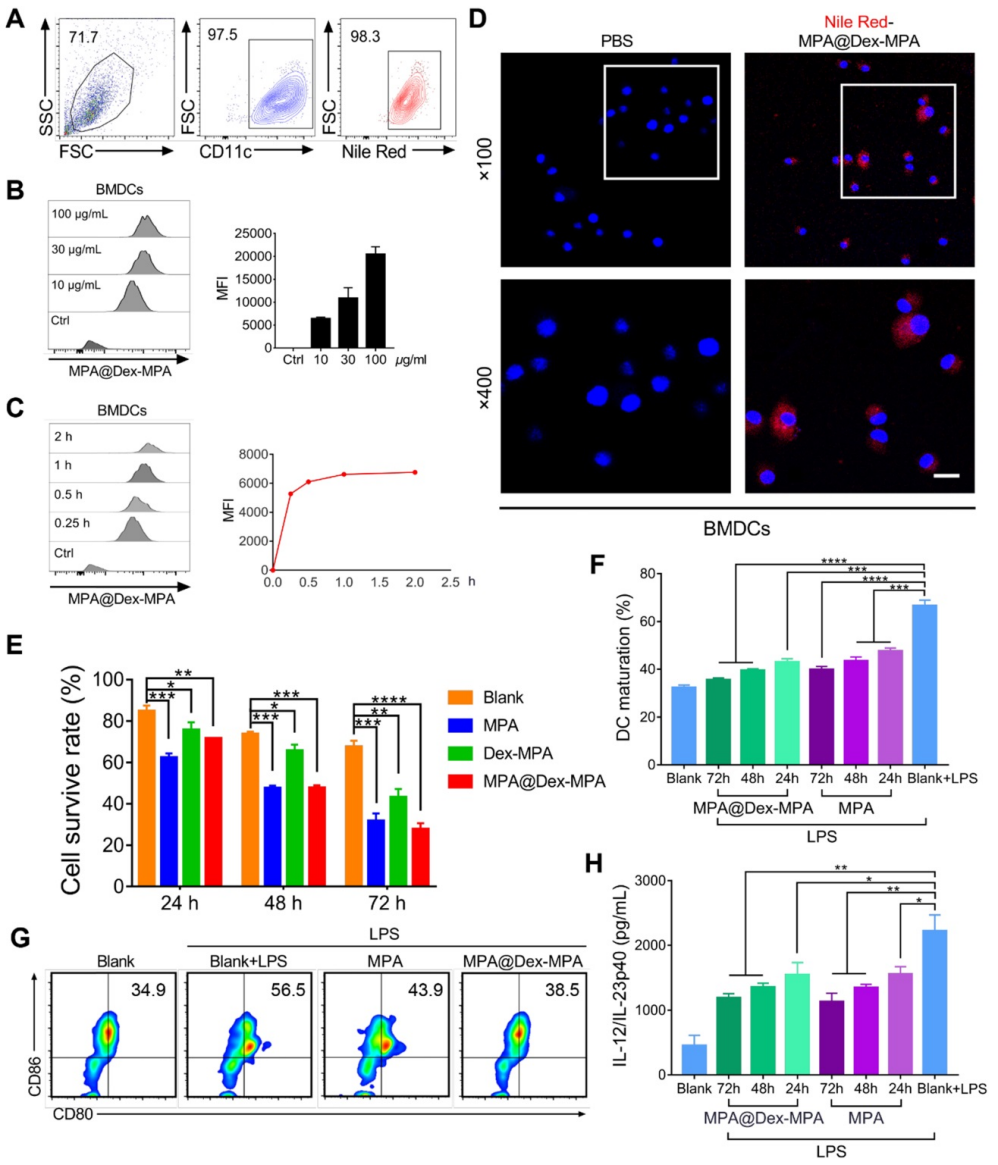
Cellular uptake of MPA@Dex-MPA NPs and the effects on viability and maturation of BMDCs by different treatments. (A-D) Internalization profiles of NR-MPA@Dex-MPA NPs into BMDCs obtained by flow cytometry. (A) Representative flow cytometry image when incubated with MPA@Dex-MPA NPs (10 μg/mL in MPA equiv.) for 15 min. (B, C) Representative flow cytometry histograms and quantification analysis for (B) indicated concentration of MPA@Dex-MPA NPs, and (C) indicated incubation time. MFI, median fluorescence intensity. (D) CLSM images of BMDCs internalized NR-MPA@Dex-MPA NPs (10 μg/mL in MPA equiv.) for 30 min. Red: Nile Red-labeled NPs; Blue: Hoechst-stained nuclei. Scale bar (20 μm) applies to all images. (E) Cell viability detected by Annexin V-FITC of BMDCs incubated with MPA, Dex-MPA NPs, or MPA@Dex-MPA NPs (10 μg/mL in MPA equiv.) for 24, 48, or 72 h. (F, G) Maturation of LPS-stimulated BMDCs after incubation with MPA@Dex-MPA or MPA (10 μg/mL in MPA equiv.) for 24, 48, or 72 h. LPS, lipopolysaccharide. (F) BMDCs maturation assessed by the ratio of CD80+, CD86+ cells among CD11c+ cells. (G) Representative flow cytometry images of BMDC maturation incubated for 48 h. (H) IL-23 secretion from BMDCs at different incubation times. IL-12/IL-23p40 levels in the supernatants were analyzed by ELISA. Data are representative of at least three independent experiments (*p < 0.05, **p < 0.01, ***p < 0.001, and ****p < 0.0001).

We also investigated the effects of MPA@Dex-MPA NPs on DCs* in vitro* (**Figure 6**). Uptake of MPA@Dex-MPA NPs by DCs was analyzed using flow cytometry by determining the intracellular fluorescence of bone marrow-derived DCs (BMDCs) incubated with various concentrations of NR-MPA@Dex-MPA NPs for different periods. The CD11c+ BMDCs displayed a high uptake level (>95% positive) at a low MPA@Dex-MPA NP concentration (10 μg/mL) for a short incubation period (15 min) (Figure 6A). The intensity of intracellular fluorescence increased gradually with incubation time or MPA@Dex-MPA concentration (Figure 6B, C), indicating that the NPs could be internalized by DCs efficiently. Confocal laser scanning microscopy (CLSM) images showed that MPA@Dex-MPA NPs were distributed to the cytoplasm of BMDCs (Figure 6D). We evaluated the cytotoxicity of MPA@Dex-MPA NPs in BMDCs. The survival rate of BMDCs decreased as incubation duration increased (Figure 6E). Less cytotoxicity was observed in BMDCs that were incubated with MPA@Dex-MPA NPs (10 μg/mL in MPA equiv.) than free MPA at the same dose for 24 h. However, comparable or even higher cytotoxicity was detected after 48 or 72 h of MPA@Dex-MPA incubation (Figure 6E). This phenomenon was attributed to the delayed release of MPA molecules from MPA@Dex-MPA NPs, consistent with the *in vitro* release profile (Figure 1E). On the contrary, Dex-MPA NPs provided lower cytotoxicity even at 72 h because of insufficient initial concentration and release rate (Figure 1E). Besides, after treatment with MPA@Dex-MPA NPs, the LPS-activated BMDCs displayed down-regulated expression of CD80 and CD86 (Figure 6F, G), as well as decreased secretion of the inflammatory cytokine IL-23 (Figure 6H), indicating marked inhibition of BMDC maturation. These findings suggested that MPA@Dex-MPA NPs not only reduced the viability of DCs, possibly by enhancing apoptosis via caspase induction 32, 66, 67, but also suppressed LPS-activated BMDC maturation.

**Figure 7:**
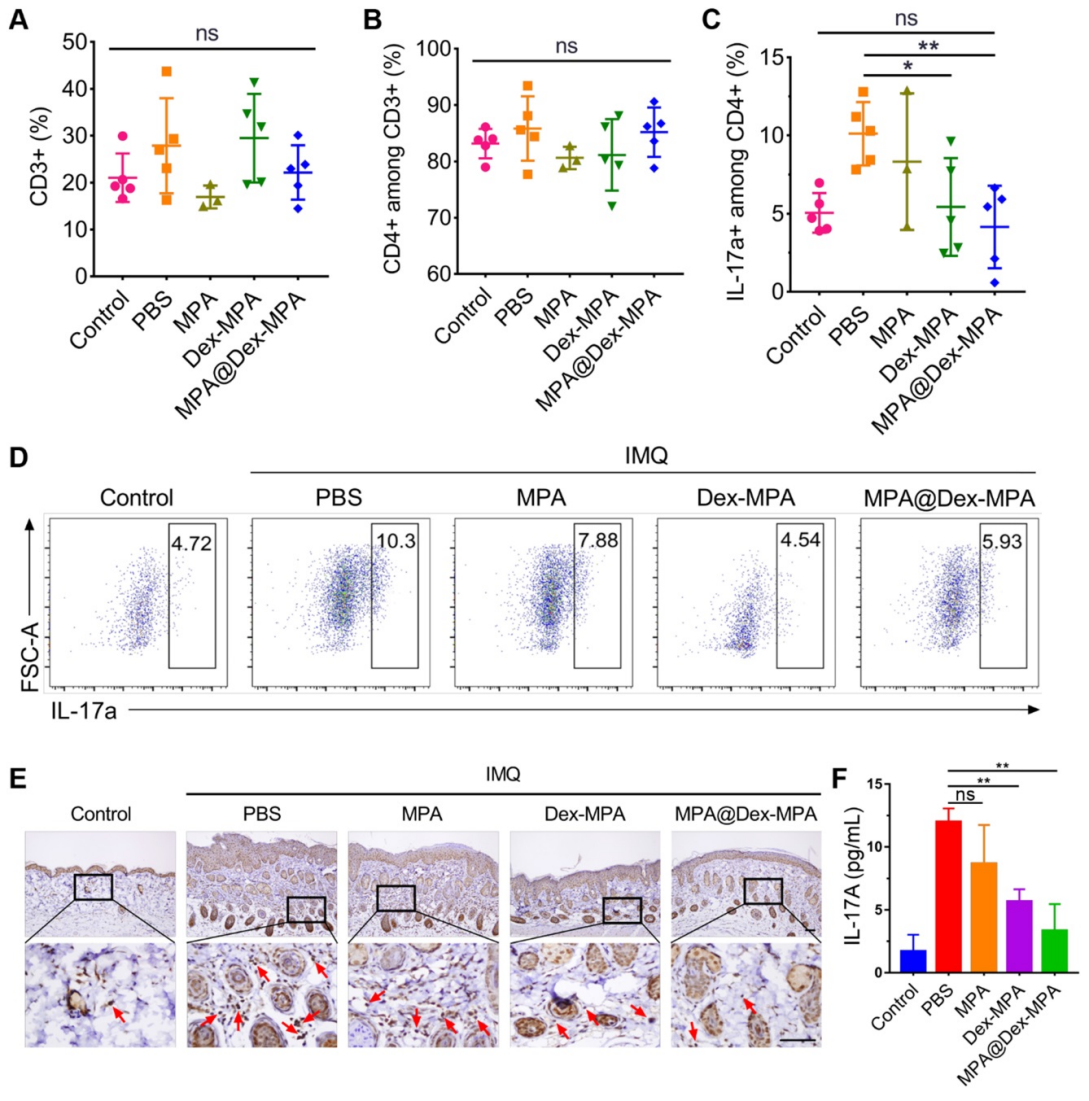
*In vivo* activation of T lymphocytes by different treatments. Populations in spleens for (A) CD3+ T cells, (B) CD3+CD4+ T cells, and (C) CD3+CD4+IL-17a+ Th17 cells; and (D) Representative flow cytometry images for CD3+CD4+IL-17a+ Th17 cells determined by flow cytometry after different treatments. (E) Representative IL-17A IHC staining images of the skin lesion following the treatments. Scale bars (100 μm) in the right images apply to the left ones. Arrows show IL-17A+ T lymphocytes. (F) Secretion of IL-17a cytokine in serum detected by ELISA following the treatments. Data are presented as means ± SEM (*n* = 5; ns, no significance, *p < 0.05, **p < 0.01; one-way ANOVA with post hoc Tukey test).

As additional evidence that the enhanced therapeutic effects of MPA@Dex-MPA NPs were mediated by declining DCs and subsequently deactivating IL-23/Th17 axis rather than the direct effect on T lymphocytes, we examined MPA@Dex-MPA treatment effects on the downstream T lymphocytes. As displayed in Figure 4, only 5.52% of MPA@Dex-MPA NPs were present in T lymphocytes, and less than 3% of T lymphocytes internalized the NPs in spleens of IMQ-treated mice, indicating that MPA@Dex-MPA NPs did not directly affect T lymphocytes. This result was also confirmed by the remarkably low cellular uptake efficiency of NR-MPA@Dex-MPA NPs by T lymphocytes* in vitro* (**Figure S6**). Quantitative flow cytometry indicated no significant differences in CD3+ and CD3+CD4+ T lymphocyte populations in spleens of all groups, attributed to the low uptake level of NPs by these cells (**Figure 7**A, B). The population of CD3+CD4+IL-17a+ Th17 cells increased significantly in spleens of PBS-treated IMQ-induced psoriasis mice due to the stimulus of high-level IL-23 secreted by activated DCs (Figures 5B and 6H). However, it was reversed to the normal level after MPA@Dex-MPA treatment (Figure 7C, D). Similar results were observed in the dLNs as well (**Figure S7**). Moreover, IL-17A cytokine secretion by T lymphocytes in dermis by IHC staining of skin lesion (Figure 7E) and serum by ELISA (Figure 7F) was reduced to a normal level after MPA@Dex-MPA treatment. We also confirmed these phenomena *in vitro* (**Figure S8**). We stimulated naïve CD4+ T cells isolated from Balb/c mice under Th17 polarizing conditions, then co-cultured them with BMDCs pretreated with MPA@Dex-MPA NPs for 48 h. The proportion of Th17 cells decreased dramatically (Figure S8), indicating that MPA@Dex-MPA could reduce the stimulatory capability of BMDCs on CD4+ T cells and prevent them from differentiating to Th17 cells.

It is well known that the activation of T lymphocytes is closely related to the activation of APCs, especially DCs. Our results indicated that IP injection of MPA@Dex-MPA NPs mediated enhanced suppression of DCs and resulted in deactivation of the downstream IL-23/Th17 axis. Considering that over-activation of DCs and IL-23/Th17 axis are vital factors in the pathogenesis of psoriasis 20, 65, our results suggested that MPA@Dex-MPA NPs attenuated the IMQ-induced skin and systemic inflammation by suppressing DCs, and subsequently deactivating the IL-23/Th17 axis. It is noteworthy that since the MPA@Dex-MPA NPs firstly decline the DCs, then deactivate the downstream immune response, rather than directly act on T cells; therefore, their indications will be not limited to psoriasis. Over-activation of DCs are the common characteristics of IMIDs, and the reversal of these over-activated DCs to normal will contribute to the treatments of IMIDs. For example, in SLE patients, the over-activated pDCs express high-level pro-inflammatory cytokines (such as TNF-α) and disrupt T/B cell tolerance, therefore inducing significant tissue lesions 16. Declining the pDCs and decreasing the TNF-α level can significantly remit the clinical symptoms 68. Similarly, in synovial tissues of RA patients, infiltration of DCs that express high-level inflammatory cytokines (such as IL-12, IL-15, IL-18, TNF-α), which played crucial roles in the development of synovium inflammatory, is commonly observed 14. A clinical trial using tolerant DC transplantation for treating RA also showed encouraging results 69. As a result, the MPA@Dex-MPA NPs may also provide options for the treatments of these DC-related IMIDs.

### Biocompatibility of MPA@Dex-MPA NPs

**Figure 8:**
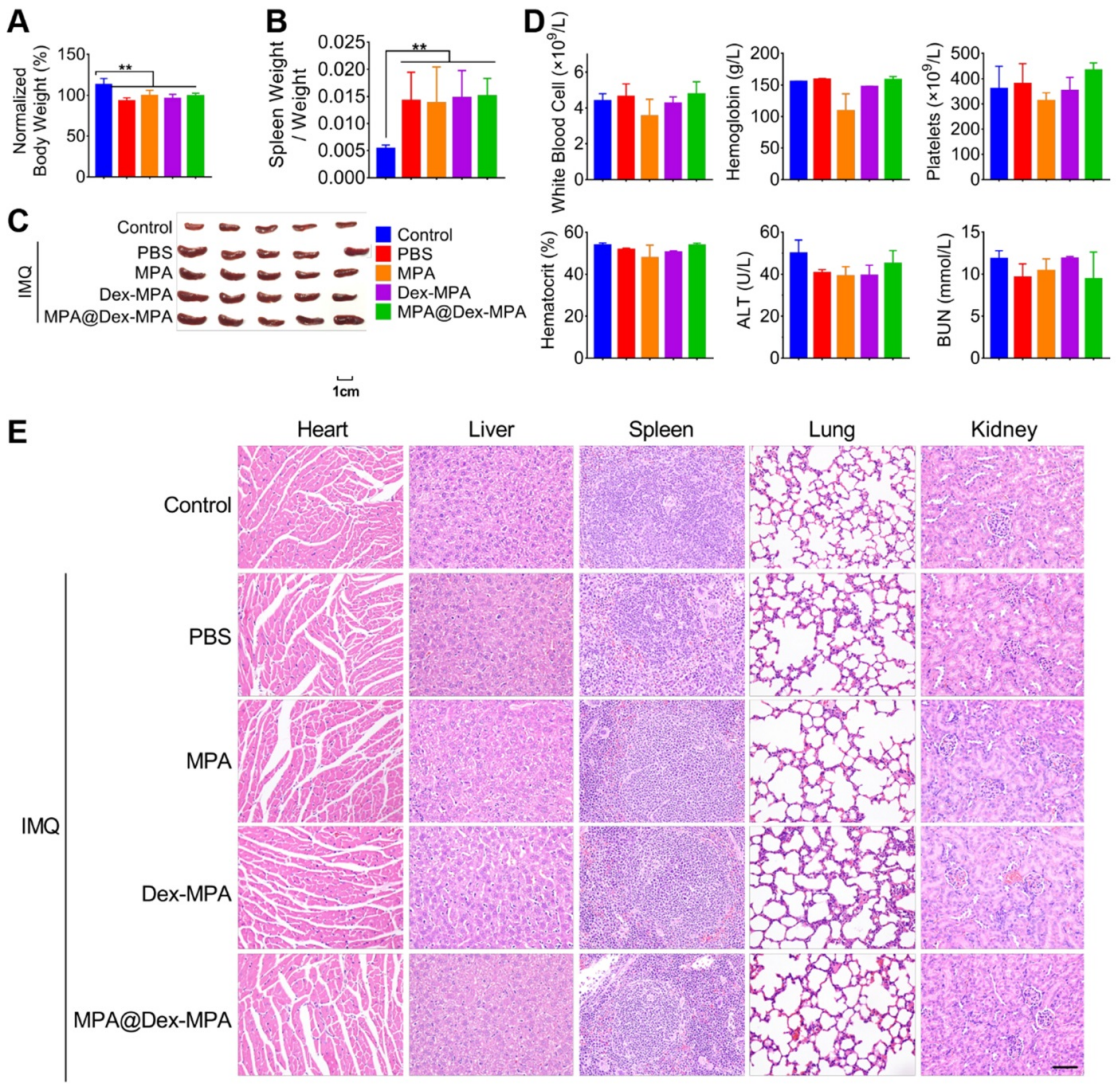
Biocompatibility of MPA@Dex-MPA NPs in Balb/c mice. Same color represents the same group in all figures. (A) Body weight on day 11 compared to that on day 0. (B, C) Changes in spleens after treatment. (B) Spleen weight/body weight ratio. (C) Phenotypic images of spleens on day 11. (D) CBCs and clinical chemistries for liver and kidney function on day 11. (E) H&E staining images of organs collected on day 11. Scale bar (50 μm) in the bottom right figure applies to the others*.* Data are presented as mean ± SEM (*n* = 5, **p < 0.01).

We evaluated the acute hematological and organ toxicity of MPA@Dex-MPA NPs in Balb/c mice. All IMQ-treated groups maintained above 90% of original body weight at the end of 9-day treatments and showed no significant difference in body weights among the groups (**Figure 8**A). Furthermore, we collected blood and organ samples (heart, liver, spleen, lung, and kidney) from these mice at the end of treatment. None of Dex-MPA NPs, MPA@Dex-MPA NPs, and MPA solution treatment reversed the splenomegaly induced by the TLR agonist effect of IMQ (Figure 8B, C). Complete blood counts (CBCs) and clinical chemistries for liver and kidney function were measured on day 11 (Figure 8D). Levels of white blood cells (WBC), platelets, hemoglobin, and hematocrit were within normal physiological ranges and showed no significant differences from the control. Besides, serum concentrations of alanine transferase (ALT) and blood urea nitrogen (BUN) in all groups were not increased (Figure 8D), indicating the MPA-based treatment did not induce acute liver or renal toxicities. Moreover, all organ samples obtained from mice treated with the MPA solution, Dex-MPA NPs, or MPA@Dex-MPA NPs showed no histopathological abnormities (Figure 8E). These results demonstrated the notable safety and biocompatibility of MPA@Dex-MPA NPs for IMQ-induced psoriasis treatment *in vivo*.

## Conclusion

We successfully prepared polysaccharide mycophenolate-based NPs, which displayed high MPA loading efficiency, low albumin binding rates, and sustained MPA release, resulting in improved pharmacokinetics *in vivo*. The MPA@Dex-MPA NPs exhibited an excellent capacity for the treatment of IMIDs (e.g., psoriasis). The MPA@Dex-MPA NPs were primarily internalized by DCs, triggered reduced viability, suppressed maturation and decreased inflammatory cytokine secretion by DCs, and deactivated the downstream IL-23/Th17 axis. Furthermore, the NPs showed good biocompatibility* in vivo*. These results demonstrated that the polysaccharide mycophenolate-based NPs are promising for the treatment of IMIDs and may be potentially useful for other DC-related diseases.

## Materials and methods

### Materials

MPA was purchased from Aladdin (Shanghai, China). 4-Dimethylaminopyridine (DMAP), 1-ethyl-3-(3-(dimethylamino)propyl)-carbodiimide hydrochloride (EDC), dextran (*M*_n_ = 10 kDa), and polyvinyl alcohol (PVA, *M*_w_ = 13-23 kDa, 87%-89% hydrolyzed) were obtained from Sigma-Aldrich (St. Louis, MO, U.S.A.). Dulbecco's modified Eagle medium (DMEM) and folic acid-free Roswell Park Memorial Institute (RPMI)-1640 were purchased from Gibco.

### Synthesis of Dex-MPA

Dex-MPA was synthesized through the esterification of MPA and dextran catalyzed by EDC/DMAP. Dextran (0.324 g, 2.0 mmol of anhydroglucose residue) was dissolved in anhydrous dimethylsulfoxide (DMSO, 50 mL) with MPA (0.640 g, 2.0 mmol). After fully dissolved, EDC (0.767 g, 4.0 mmol) and DMAP (0.293 g, 2.4 mmol) were added, and the solution was allowed to react for 24 h. The reaction was monitored by TLC, and the resultant Dex-MPA was precipitated by water, followed by centrifugation (5000 rpm, 5 min). The crude product was purified by repetitive resuspension and filtration, and the final product was obtained by lyophilization as a white solid. ^1^H NMR was performed on a Bruker AV400 NMR spectrometer (Billerica, MA, U.S.A.).

### Preparation and characterization of NPs

An ultrasonic emulsification and solution evaporation method was employed to prepare Dex-MPA NPs and MPA@Dex-MPA NPs. Dex-MPA in CHCl_3_ (10 mg/mL, 5 mL) and PVA aqueous solution (3 mg/mL, 50 mL) were mixed, and the mixture was emulsified with a probe sonicator (25% power, using a (10 s)-(5 s) on-off program for 5 min). CHCl_3_ was removed by complete evaporation under aseptic conditions, and PVA was removed by repetitive centrifugation (12,000 rpm, 10 min) and resuspension for three times. For preparing MPA@Dex-MPA NPs, 15 mg of extra free MPA was incorporated into the Dex-MPA solution. The MPA content in Dex-MPA NPs or MPA@Dex-MPA NPs was determined by a Shimadzu 1800 UV-vis spectrometer (Tokyo, Japan) using a standard curve. TEM was performed using a Hitachi TM4000Plus TEM, 100 kV (Tokyo, Japan), and DLS was performed on a Malvern Nano-ZS90 zetasizer (Malvern, U.K.). Serum protein adsorption was performed using BSA as the model protein by BCA assay. BSA (160 µg/mL) was incubated with MPA@Dex-MPA NPs at various concentrations (320, 160, 80, and 40 µg/mL) for different periods (0.5, 1, 2, 4, 8, and 12 h). The NPs were removed through centrifugation (10,000 rpm, 15 min), and the concentration of BSA in the supernatant was determined to calculate the retention of BSA and amount of absorbed BSA per unit weight of NPs (*q*_e_) by the following equations:


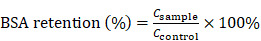
(Eq. 1)


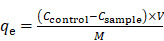
(Eq. 2)

where *C*_control_ and *C*_sample_ represent concentrations of BSA without or with incubation of NPs, respectively, *V* represents the solution volume, and *M* represents the NPs mass.

### *In vitro* MPA release

The release profile of MPA was performed by a dialysis method. Briefly, dispersion of NPs (10 mg/mL, 2 mL) was added into a dialysis tube (MWCO = 7000 Da), and the NPs were dialyzed against 20 mL of phosphate-buffered saline (PBS, pH = 7.4) containing 0.02% of Tween 80 at 37 °C with continuous shaking at 100 rpm. Two mL of the solution were harvested daily to determine the MPA concentration through UV-vis spectra, and an equal volume of fresh solution was added.

### Mice

Seven-week-old female Balb/c mice and eight-week-old female Sprague-Dawley (SD) rats were purchased from Liaoningchangsheng Bio Co. Ltd. (Liaoning, China). All animals were maintained in specific pathogen-free conditions in the Animal Center of Huazhong University of Science and Technology. During the experiments, the animals were sustained at 25 ± 1 °C and 60% ± 10% humidity and exposed to alternate 12 h cycles of light and darkness. All animal studies were performed according to Chinese laws and the local Ethical Committee Quantita Protocol.

### IMQ-induced psoriasis mouse model and *in vivo* treatment

Mice were topically treated with 62.5 mg of 5% IMQ cream (MED-SHINE Pharmaceutical Co., Sichuan, China) daily on the shaved back skin for ten consecutive days to establish the psoriasis mouse model. Mice were randomly divided into five groups. From the second day of IMQ treatment, mice in the model groups were IP injected daily with MPA@Dex-MPA NPs, Dex-MPA NPs, free MPA solution (10 mg/kg in MPA equiv.), or an equal volume of PBS (100 μL) for 9 days. All therapeutics were applied under anesthesia with tiletamine/zolazepam (30 mg/kg) and xylazine (10 mg/kg). Mice were sacrificed on day 11, and full-thickness skin samples of identical areas were harvested. The spleen was obtained and weighed, and inguinal lymph nodes were collected. A clinical assessment of the severity of psoriatic skin lesions was performed daily using Psoriasis Area and Severity Index (PASI) scores, which rates erythema, scaling, and thickness on a scale of 0 to 4 as follows: 0, no symptoms; 1, mild; 2, moderate; 3, severe; and 4, very severe 70, 71.

### Histological evaluation

Skin samples were fixed with 4% paraformaldehyde (PFA), embedded in paraffin, and sectioned in 5 μm thickness. Tissue sections were stained with H&E using a standard protocol and then analyzed on an Eclipse Ni-E upright microscope (Nikon Corporation, Tokyo, Japan). Epidermal thickness was measured based on at least 50 hand-drawn line segments connecting dermal-epidermal junction (DEJ) to SC per section using NIS-elements microscopic imaging software (Nikon). For immunohistochemistry, skin sections were deparaffinized in xylene and rehydrated in distilled water. Antigen retrieval was performed by immersing slides in boiling sodium citrate buffer (10 mM sodium citrate, pH 6.0) for 10 min. Thereafter, slides were placed in an aqueous sodium hydroxide solution (1%) and hydrogen peroxide (1%) for 20 min to quench endogenous peroxidase activity. Nonspecific binding was blocked by Tris buffer containing BSA (3%) and Triton X-100 (0.2%) at room temperature for 1 h. Subsequently, sections were incubated with anti-Ki-67 antibody (1:500; Abcam), anti-CD11c antibody (1:200; NOVUS BIOLOGICALS), or anti-IL-17 antibody (1:500; Abcam) at 4 °C for 24 h. Slides were then washed and incubated with biotinylated goat antirabbit secondary antibody (1:200; Vector Laboratories) at room temperature for 1 h. All slides were rinsed and incubated with Vectastain Elite ABC kit (Vector Laboratories) for 30 min, followed by incubation with DAB substrate (Vector Laboratories) for 5 min. Finally, slides were counterstained with hematoxylin and examined under a microscope. CD11c-positive areas were expressed as percentages of the total area of the image. Areas in five random sections per mouse were quantified using ImageJ software.

### *In vivo* cellular internalization profile

After establishing IMQ-induced skin inflammation, Balb/c mice (*n* = 5 for each time point) were given Nile Red-labeled MPA@Dex-MPA NPs (10 mg/kg MPA loaded) via IP injection. The animals were euthanized and perfused at 2 h post-administration. Spleens were harvested and processed to obtain single-cell suspensions as described above. These cells were labeled with the following antibodies: PerCP-Cy5.5-labeled anti-mouse CD3e, FITC-labeled anti-mouse CD19, PE-Cy7-labeled CD11c, and APC-labeled anti-mouse F4/80 (eBioscience Inc., San Diego, CA, U.S.A.). The cells were then analyzed using a BD LSR II Green flow cytometer with FlowJo software (TreeStar, San Carlos, CA, U. S. A.).

### Analysis of serum cytokine levels

Mice serum samples were collected on day 11 before sacrifice. Levels of cytokines were assessed by ELISA according to the manufacturer's protocols. Total serum IL-23 levels were measured using a commercial mouse IL-12/IL-23 p40 ELISA kit (DAKEWE, Shenzhen, China).

### Flow cytometry analysis

Single-cell suspensions from spleens and lymph nodes were prepared. Briefly, spleens or inguinal lymph nodes were collected and passed through a cell strainer (pore size: 70 μm) to obtain single-cell splenocytes suspensions, and red blood cells (RBC) were removed with RBC lysis buffer (BioLegend, San Diego, CA, U. S. A.). Nonspecific antibody binding was blocked with an anti-CD16/32 antibody (BD Biosciences, San Jose, CA, U. S. A.), and then cells were stained for surface antigens. For intracellular staining of IL-17A and Foxp3, cells were fixed after surface staining and permeabilized with Cytofix/Cytoperm solution (BD Biosciences) and stained intracellularly. Samples were examined on a BD LSRFortessa cell analyzer (BD Biosciences), and data were analyzed with FlowJo software.

### Acquisition of BMDCs

Bone marrow was extracted from femurs and tibias of a mouse by flushing each shaft with RPMI-1640 culture medium (Gibco, Waltham, MA, U. S. A.) using a syringe. After removal of RBCs by RBC lysis buffer, bone marrow cells were suspended, filtered through a cell strainer (pore size: 70 μm), and resuspended in a complete RPMI culture medium containing 10 ng/mL of granulocyte-macrophage colony-stimulating factor (GM-CSF; CreaGene, Seongnam, Korea) and IL-4 (CreaGene). Cells were seeded in 6-well plates at 1 × 10^7^ cells/well. The medium was replaced every two days, and nonadherent and loosely adherent cells were collected and replated after six days for further experiments.

### *In vitro* cellular uptake assay and stimulation of BMDC

BMDCs (5 × 10^5^ cells/well) were seeded in 24-well plates and cultured in FA-containing RPMI 1640 medium. Then, the cells were treated with Nile-Red-labeled MPA@Dex-MPA NPs at different concentrations for indicated periods. Cells were collected for cytometry analysis, as described above. For CLSM imaging, cells were fixed and incubated with Hoechst 33342 before examining under a CLSM (Olympus FV500, Japan). For experiments in which BMDCs were stimulated with a TLR4 ligand, cells were first treated with PBS, free MPA solution, or MPA@Dex-MPA NPs (10 μg/mL in MPA equiv.) for 24, 48, or 72 h, and then cultured in the presence of LPS (resiquimod; Sigma-Aldrich, St. Louis, MO, U.S.A. 50 ng/mL) for 18 h. Cells were collected to detect CD80 and CD86 expression by flow cytometry, and supernatants were collected for IL-12/IL-23p40 levels determination by ELISA.

### *In vitro* differentiation of Th17 cells

Naïve CD4+ T cells were purified from spleens of Balb/c mice using a mouse naïve CD4+ T cell isolation kit (Miltenyi Biotec) and higher than 98% purity was validated by flow cytometry. Next, purified naïve CD4+ T cells were stimulated with plate-bound anti-CD3 (2 μg/mL, Biolegend) and anti-CD28 (2 μg/mL, Biolegend) for 3 days under Th17 differentiation conditions: IL-6 (30 ng/mL, Biolegend), TGF-β (5 ng/mL, Biolegend), anti-IL-4 (10 μg/mL, Biolegend), anti-IFN-γ (10 μg/mL, Biolegend) and IL-23 (20 ng/mL, Biolegend).

### Blood concentration of MPA *in vivo*

Eight-week-old Sprague-Dawley (SD) Rats were randomly separated into two groups (five rats per group), and then were IP injected with MPA solution or MPA@Dex-MPA NPs (10 mg/kg in MPA equiv.). For each group, 250-300 μL of blood was collected from jugular vein at 0, 1, 2, 4, 8, 24, 48, 72, 120, and 168 h post-injection using a disposable syringe (1 mL, 26G, Korea Vaccine, Seoul, South Korea) containing sodium heparin (100 IU/mL).

### Toxicology studies

Blood samples of all groups were collected on day 11 before sacrifice. Some of the blood was collected in EDTA spray-coated tubes and analyzed immediately by CBC on a Hemavet blood counter (Drew Scientific). Serum concentrations of ALT and BUN were measured using reagents from Teco Diagnostics. Hearts, livers, spleens, lungs, and kidneys were also harvested from each group and fixed in a 4% PFA solution. The organs were embedded in paraffin, sectioned, and processed for H&E staining. The H&E sections were imaged on a Nikon Ni-E (Nikon, Minato, Tokyo, Japan).

### Statistical analysis

Experiments were repeated three times, and data were expressed as means ± SEM unless otherwise noted. Differences among groups were analyzed by one-way analysis of variance (ANOVA), followed by the post hoc Tukey test. All statistical analyses were performed using SPSS Statistics 20 software (SPSS Inc.), and *p* values < 0.05 were considered statistically significant.

## Supplementary Material

Supplementary figures.

## Figures and Tables

**Table 1:** Characterization of the NPs.

Entry	Total MPA loading content *^a^*	Conjugated MPA	Encapsulated MPA	Loading efficiency of encapsulated MPA
Dex-MPA	63.51%	63.51%	-	-
MPA@Dex-MPA	70.99%	50.49%	20.50%	85.95%

*^a^* Determined by UV-vis spectroscopy.

**Table 2:** Pharmacokinetic parameters of MPA and MPA@Dex-MPA NPs.

Pharmacokinetic parameters of MPA	MPA	MPA@Dex-MPA NPs
AUC (µg·h/mL)	79.8 ± 8.8	23.2 ± 0.8
AUC_0-24h_ (µg·h/mL)	77.9 ± 8.8	18.4 ± 0.6
Terminal half-life *t*_1/2_ (h)	14.7 ± 1.0	24.6 ± 0.7
*C*_max_ (µg/mL) ^a)^	11.5 ± 2.1	3.2 ±0.1
*T*_max_ (h) ^a)^	1	1

^a^ Plasma concentration in 0-1 h was not detected. AUC: total area under the plasma concentration-time curve; AUC_0-24h_: area under the plasma concentration-time curve from 0 to 24 h; *C*_max_: peak plasma concentration; *T*_max_: time to attain C_max_. Values expressed as Mean ± SEM.

## Abbreviations

IMIDs: immune-mediated inflammatory diseases
MPA: mycophenolic acid
MMF: mycophenolate mofetil
NPs: nanoparticles
DCs: dendritic cells
IL-23: Interleukin-23
IMPDH: inosine 5'-monophosphate dehydrogenase
PLGA: poly(lactic-co-glycolic acid)
IMQ: imiquimod
IP injection: intraperitoneal injection
PASI: psoriasis area and severity index
H&E: hematoxylin and eosin
IHC: immunohistochemistry
Dex-MPA: Dextran-MPA conjugate
TEM: transmission electron microscope
DLS: dynamic laser scattering
*D*_h_: hydrodynamic diameter
BSA: bovine serum albumin
pDCs: plasmacytoid DCs
BMDCs: bone marrow-derived DCs
dLN: draining lymph node
ELISA: enzyme-linked immunosorbent assay
CBCs: complete blood counts
WBC: white blood cell
ALT: alanine transferase
BUN: blood urea nitrogen
EDC: 1-ethyl-3-(3-(dimethylamino)propyl)-carbodiimide hydrochloride
DMAP: 4-dimethylaminopyridine
PVA: polyvinyl alcohol
DMEM: Dulbecco's modified Eagle medium
DMSO: dimethylsulfoxide
CLSM: confocal laser scanning microscopy
