# Supplementary material for: Polysaccharide mycophenolate-based nanoparticles for enhanced immunosuppression and treatment of immune-mediated inflammatory diseases
Source: Theranostics. 2021 Jan 26;11(8):3694–709. doi: 10.7150/thno.52891 (PMC7914372; doi:10.7150/thno.52891)
Supplement: Supplementary file 1 — Supplementary figures. [file thnov11p3694s1.pdf]

Supporting Information for

Polysaccharide mycophenolate-based nanoparticles for enhanced immunosuppression and treatment of immune-mediated inflammatory diseases

Yuce Li[#], Yuchen Lou[#], Yu Chen, Jing Yang, Danqi Li, Biling Jiang, Jiajia Lan, Jingjing Wen, Yangxue Fu, Yamin Zhang, Juan Tao, Jintao Zhu**

Table of contents:

Figure S1. ^{1}H NMR of MPA and Dex-MPA.

Figure S2. UV–vis spectra of MPA and Dex-MPA.

Figure S3. Protein adsorption of MPA@Dex-MPA NPs.

Figure S4. HPLC-MS results for neat MPA and hydrolyzed Dex-MPA products.

Figure S5. Distribution of MPA@Dex-MPA NPs in different cell subsets in spleens of healthy Balb/c mice.

Figure S6. Internalization of MPA@Dex-MPA NPs by T cells *in vitro*.

Figure S7. Population of Th17 cells in the draining lymph nodes after different treatments.

Figure S8. *In vitro* investigation of MPA@Dex-MPA NPs on the stimulatory capability of BMDCs on CD4+ T cells differentiating to Th17.

Supporting Figures:

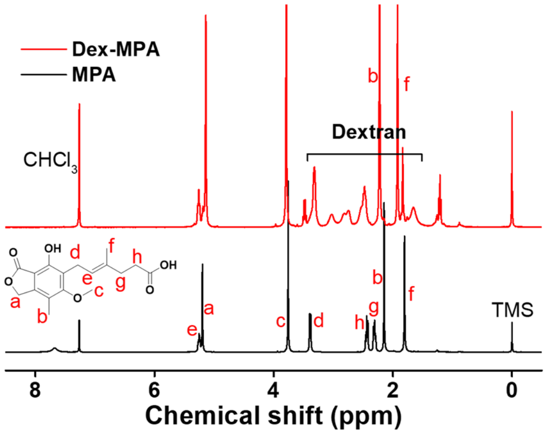

Figure S1. ^{1}H NMR of MPA and Dex-MPA in $CDCl_3$.

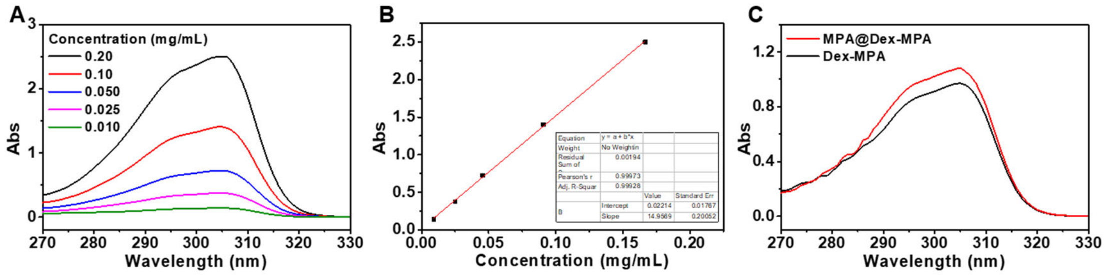

Figure S2. UV–vis spectra of MPA and Dex-MPA. (A) UV–vis spectra of MPA at different concentrations. (B) Standard curve obtained by Abs of MPA at 305 nm. (C) UV–vis spectra of Dex-MPA and MPA@Dex-MPA at 0.1 mg/mL.

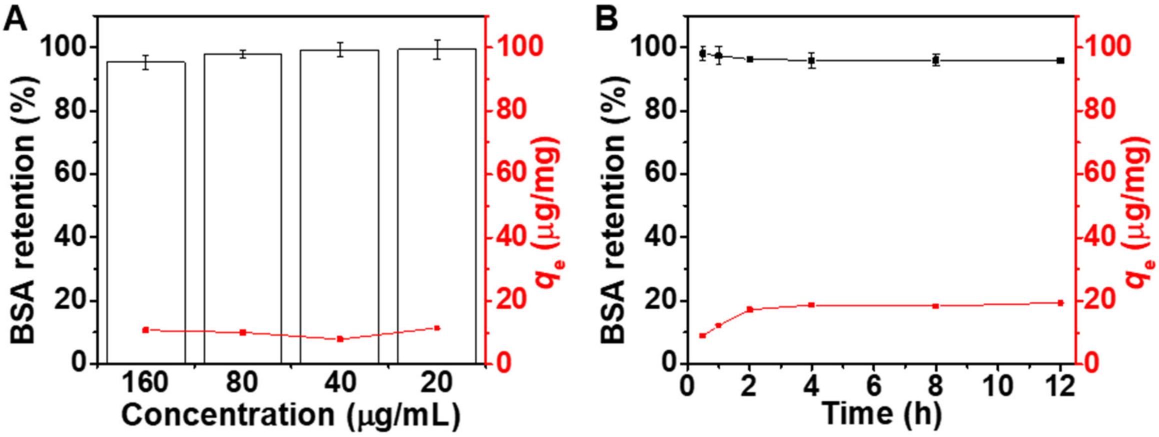

Figure S3. Protein adsorption of MPA@Dex-MPA NPs incubated at different concentrations of NPs (A) for different periods (B). The retention of BSA in the supernatant and amounts of absorbed BSA per unit weight of NPs (q_e) were calculated by the concentrations of supernatant BSA.

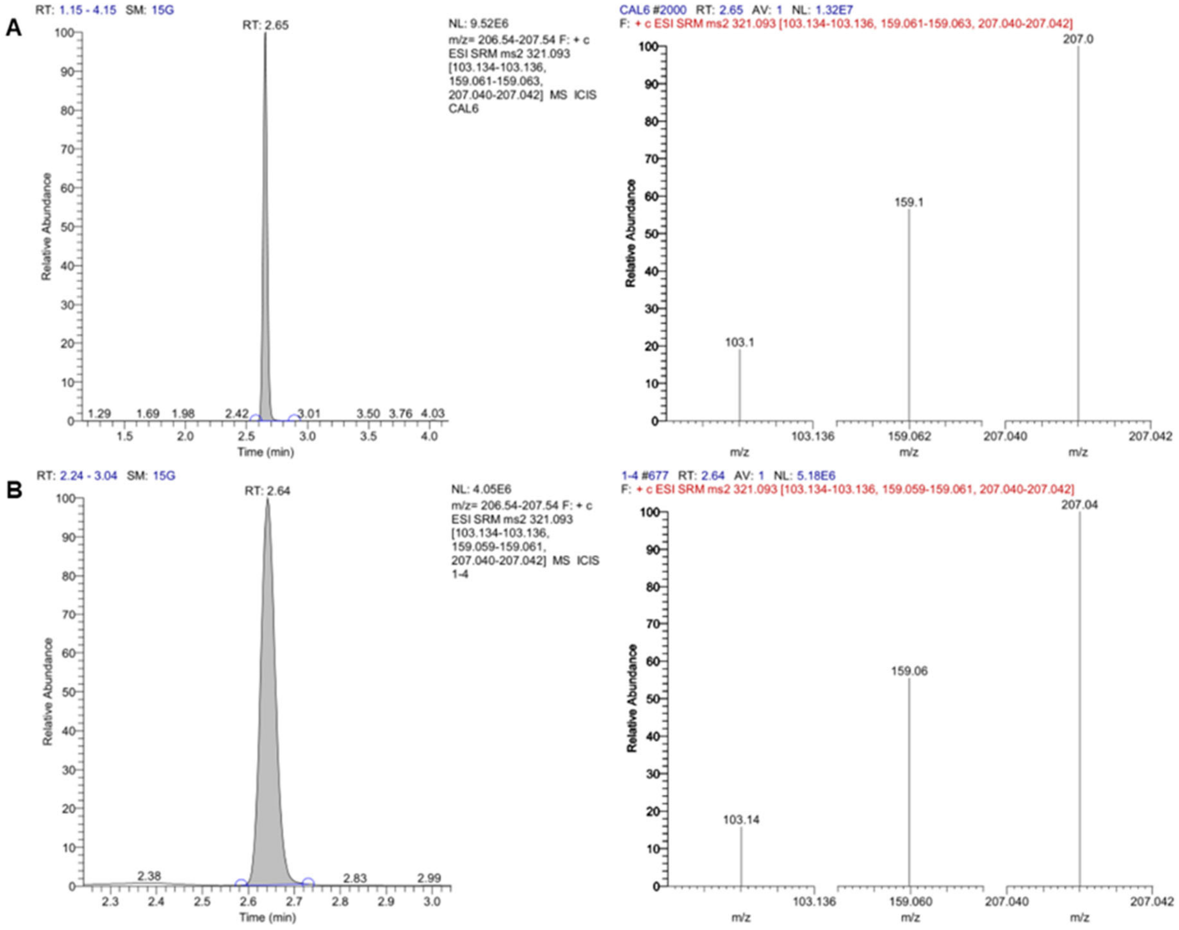

Figure S4. HPLC-MS results for (a) neat MPA and (b) hydrolyzed Dex-MPA products.

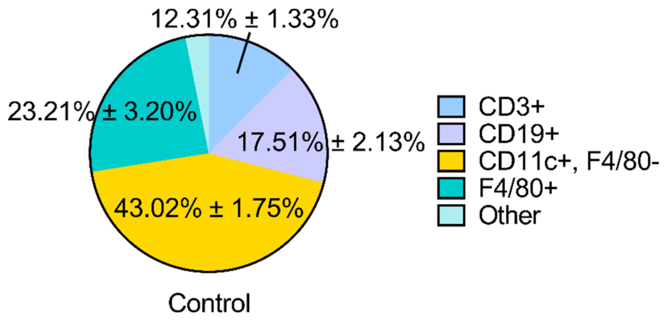

Figure S5. Distribution of MPA@Dex-MPA NPs in different cell subsets in spleens of healthy Balb/c mice. Results are expressed as mean ± SD. CD3+, T cells; CD19+, B cells; CD11c+, F4/80−, DCs; F4/80+, macrophages.

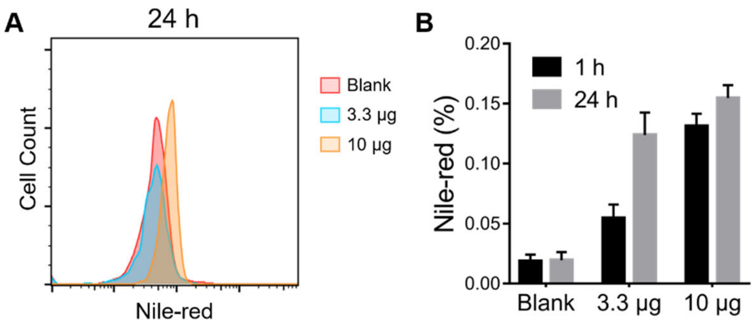

Figure S6. Internalization of MPA@Dex-MPA NPs by T cells *in vitro*. CD4+ T cells were isolated from spleens of Balb/c mice and cultured *in vitro*, and incubated with Nile-Red-labeled MPA@Dex-MPA NPs. Flow cytometry was performed to detect Nile-Red positive CD4+ cells. (A) Representative flow cytometry histogram and (B) quantification analysis for the indicated concentrations of MPA@Dex-MPA.

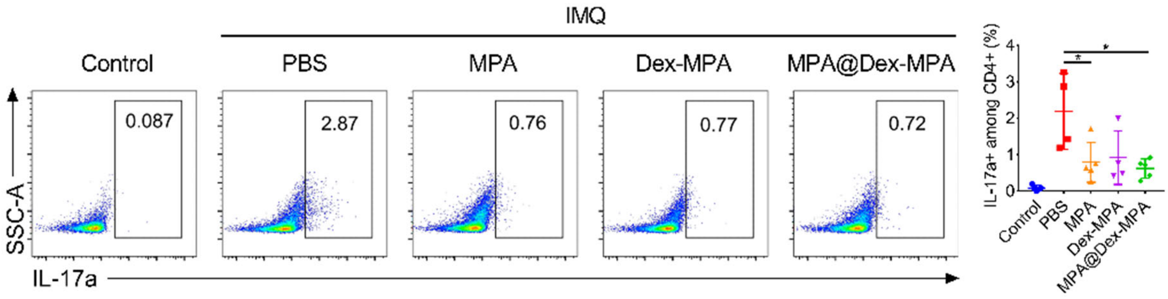

Figure S7. Populations of Th17 cells in the draining lymph nodes after different treatments. Percentage of CD3+CD4+IL-17a+ Th17 cells in inguinal lymph nodes determined by flow

cytometry. Data are presented as means ± SEM ($n = 5$ mice/group; *$p < 0.05$; one-way ANOVA with post-hoc Tukey test).

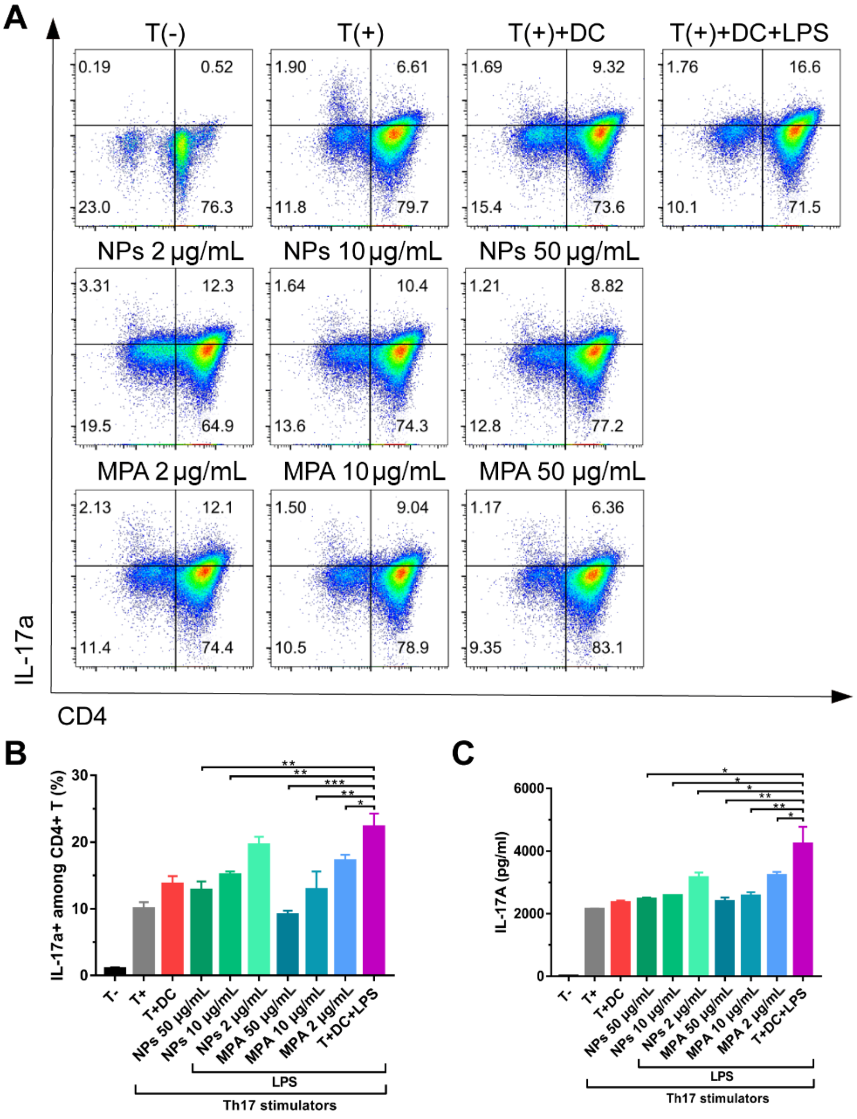

Figure S8. *In vitro* investigation of MPA@Dex-MPA NPs effect on the stimulatory capability of BMDCs on CD4+ T cells differentiating into Th17. BMDCs were incubated with MPA@Dex-MPA NPs or MPA (2, 10 or 50 μg/mL) for 48h, stimulated with LPS (100 ng/mL) for 18 h. Then, these BMDCs were co-cultured with CD4+ T cells isolated from Balb/c mice (at a ratio of 2×10^5 BMDCs to 1×10^6 CD4+ T cells) for 3 days, and treated with (+) or without (−) Th17 differentiation stimulators. (A, B) CD4+IL-17a+ T cells were detected by flow cytometry and (C) the supernatant was collected for ELISA. Data are representative of at least three independent experiments (*$p < 0.05$, **$p < 0.01$ and ***$p < 0.001$).
